# Characterization and Comparison of the 10-2 SITA-Standard and Fast Algorithms

**DOI:** 10.1100/2012/821802

**Published:** 2012-05-02

**Authors:** Yaniv Barkana, Erez Bakshi, Yakov Goldich, Yair Morad, Audrey Kaplan, Isaac Avni, David Zadok

**Affiliations:** Department of Ophthalmology, Assaf Harofeh Medical Center, Zerifin 73000, Israel

## Abstract

*Purpose*: To compare the 10-2 SITA-standard and SITA-fast visual field programs in patients with glaucoma. *Methods*: We enrolled 26 patients with open angle glaucoma with involvement of at least one paracentral location on 24-2 SITA-standard field test. Each subject performed 10-2 SITA-standard and SITA-fast tests. Within 2 months this sequence of tests was repeated. *Results*: SITA-fast was 30% shorter than SITA-standard (5.5 ± 1.1 vs 7.9 ± 1.1 minutes, *P* < 0.001). Mean MD was statistically significantly higher for SITA-standard compared with SITA-fast at first visit (Δ = 0.3 dB, *P* = 0.017) but not second visit. Inter-visit difference in MD or in number of depressed points was not significant for both programs. Bland-Altman analysis showed that clinically significant variations can exist in individual instances between the 2 programs and between repeat tests with the same program. *Conclusions*: The 10-2 SITA-fast algorithm is significantly shorter than SITA-standard. The two programs have similar long-term variability. Average same-visit between-program and same-program between-visit sensitivity results were similar for the study population, but clinically significant variability was observed for some individual test pairs. Group inter- and intra-program test results may be comparable, but in the management of the individual patient field change should be verified by repeat testing.

## 1. Introduction

There is a continuing search for the ideal visual field test that will be as short as possible with minimal test-retest variability. The Swedish interactive threshold algorithms (SITAs) have been shown to achieve a 50–70% reduction in test duration in comparison to the full threshold algorithms, without sacrificing accuracy [1–4]. Two SITA algorithms are currently available and in widespread use, SITA-standard and SITA-fast. The SITA-standard algorithm uses a 4-2 dB step size and the SITA-fast algorithm only a 4 dB step size. The characteristics of these algorithms, such as test duration, inter visit variability and inter algorithm differences (standard versus fast) should be known for the clinician to optimally analyze test results, compare results obtained by the two algorithms, and distinguish glaucomatous change from test variability. Studies of these characteristics have been reported using the 30-2 programs [3–7]. These can likely be extrapolated for the interpretation of the commonly used and similar 24-2 programs.

However, when the glaucomatous visual field loss threatens or involves the central vision, it is advisable to also use the central 10-2 program for diagnosis and monitoring [8, 9]. Therefore, it is important to know the characteristics of both 10-2 SITA testing algorithms and whether they can be used interchangeably.

The purpose of this study was to characterize and compare the properties of the 10-2 SITA-standard and SITA-fast visual field algorithms in patients with glaucoma.

## 2. Methods

The study was approved by the Institutional Review Board of the Assaf Harofeh Medical Center and adhered to the tenets of the Declaration of Helsinki, and written informed consent was obtained from all participants. Glaucoma patients with field loss involving central vision were recruited from the glaucoma outpatient clinic at the Assaf Harofeh Medical Center. Involvement of central vision was determined if on 24-2 SITA-standard examination one of the central 4 points had reduced sensitivity on the pattern deviation plot with *P* < 0.5%. It follows that all study participants had previous experience with automated perimetry, although not necessarily with the 10-2 program.

Exclusion criteria were corrected visual acuity in the enrolled eye 20/100 or worse, spherical equivalent refractive error ≥ 6D or astigmatism ≥ 3D, and any eye disease other than glaucoma or any neurological disease that may affect the results of automated perimetry.

Only one eye of each patient was included in this study. All patients performed 10-2 SITA-standard and SITA-fast tests of the same eye, always in this order, separated by a rest period of at least 15 minutes. The two tests were repeated on a different day within a 2-month period. In all examinations near correction was provided and the standard fixation target and test stimulus size III were used.

Reliability parameters, test duration, and sensitivity results were compared between the two programs during each visit and between the test pairs with the same program during both visits. Sensitivity results were quantified by both mean deviation (MD), and the total number of points depressed at *P* < 1% or *P* < 2% on the pattern deviation plot.

The Kolmogorov-Smirnov test was used to check whether visual field parameters were normally distributed. The 2-tailed, paired *t*-test was used to compare normally distributed parameters and the Wilcoxon signed ranks test for the nonnormally distributed parameters. Since for each parameter there were two paired comparisons, the Bonferroni-adjusted significance level was 0.025.

 In addition, we used the Bland-Altman method in order to determine the level of agreement between the 10-2 SITA algorithms in assessing visual field sensitivity. When comparing 2 measurement methods in clinical practice, it is needed to know the degree of agreement between them and thus whether they are interchangeable. Bland and Altman proposed that the use of correlation coefficients for this purpose may be misleading and inappropriate and suggested an alternative approach [10]. The Bland-Altman method assesses the agreement between two measurement methods by plotting, for each subject, the intermethod difference against the average of the two measurements and generating the summary statistic “95% limits of agreement” (LoA), that is, mean ± 1.96 SD of the differences. This statistic provides for the user of the studied measurement methods the range of differences that can be expected in 95% of cases, that is, most cases in everyday use excluding the most atypical outliers.

## 3. Results

Twenty-six eyes of 26 patients were included in the study. There were 15 women and 11 men, with a mean age of 68.9 ± 10.1 years (range 44 to 81 years). Mean LogMAR corrected visual acuity in the tested eyes was 0.18 ± 0.15. Mean deviation on the 24-2 field used for inclusion in the study was −15.56 ± 8.06.

Test duration of SITA-fast was 30% shorter than SITA-standard. Average duration for the two algorithms was 5.5 ± 1.1 and 7.9 ± 1.1 minutes, respectively (*P* < 0.001).

Tables 1 and 2 show sensitivity and reliability parameters, respectively, for each of the 4 tests, demonstrating the inter- and intraalgorithm variability. When mean MD was compared, there was no statistically significant intervisit difference for both programs. The Intravisit inter program difference in mean MD was statistically significant during the first visit but not the second visit. When we compared the number of depressed points at *P* < 1% or *P* < 2% on the pattern deviation plot, there were no statistically significant differences in intervisit intra program or Intravisit inter program results.

There were no statistically significant differences in any reliability parameter for all test pairs.

**Figure 1:**
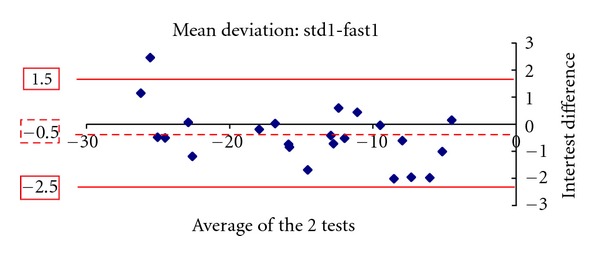
The Bland-Altman plot showing agreement in mean deviation between the 10-2 SITA-standard and SITA-fast tests during the first visit. The dashed line marks the average difference between the two algorithms. The solid lines mark the upper and lower limits of the 95% limits of agreement.

**Figure 2:**
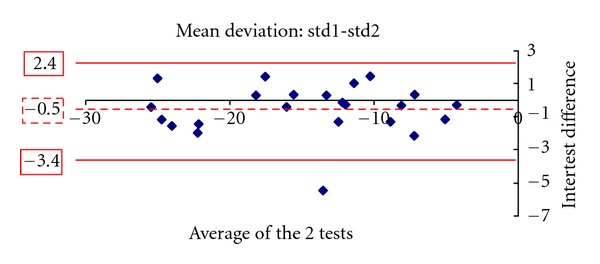
The Bland-Altman plot showing agreement in mean deviation between the first and second visit 10-2 SITA standard tests. The dashed line marks the average difference between the two algorithms. The solid lines mark the upper and lower limits of the 95% limits of agreement.

**Figure 3:**
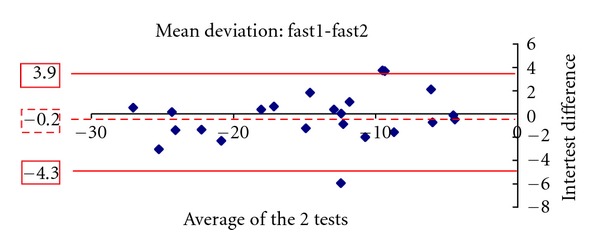
The Bland-Altman plot showing agreement in mean deviation between the first and second visit 10-2 SITA fast tests. The dashed line marks the average difference between the two algorithms. The solid lines mark the upper and lower limits of the 95% limits of agreement.

**Figure 4:**
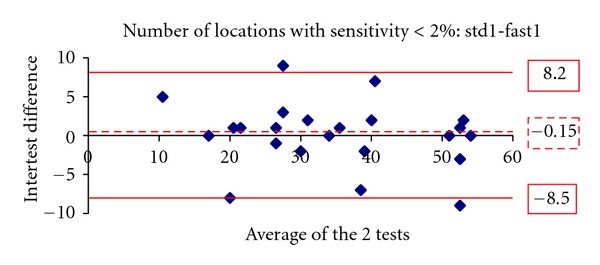
The Bland-Altman plot showing agreement in number of depressed points at least at *P* < 2% between the first visit 10-2 SITA-standard and SITA-fast tests. The dashed line marks the average difference between the two algorithms. The solid lines mark the upper and lower limits of the 95% limits of agreement.

**Figure 5:**
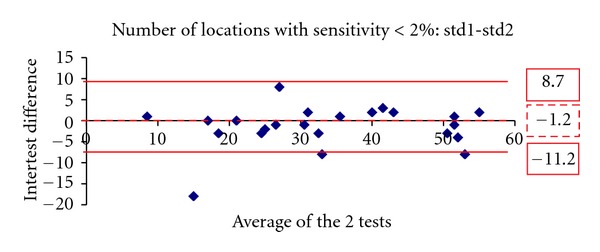
The Bland-Altman plot showing agreement in number of depressed points at least at *P* < 2% between the first and second visit 10-2 SITA-standard tests. The dashed line marks the average difference between the two algorithms. The solid lines mark the upper and lower limits of the 95% limits of agreement.

**Figure 6:**
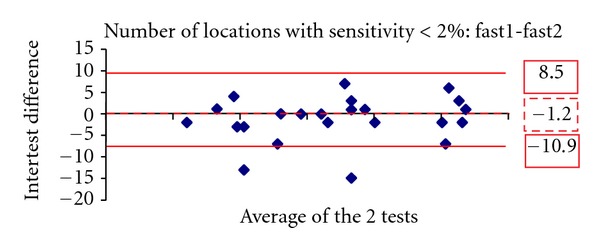
The Bland-Altman plot showing agreement in number of depressed points at least at *P* < 2% between the first and second visit 10-2 SITA fast tests. The dashed line marks the average difference between the two algorithms. The solid lines mark the upper and lower limits of the 95% limits of agreement.

Figures 1, 2, 3, 4, 5 and 6 show the Bland-Altman plots evaluating intra- and interprogram agreement in MD values and number of depressed points. These graphs demonstrate clearly that even though the average difference between measurements was nearly zero, considerable variability can exist, both between the two programs and between visits using the same program. This can be appreciated visually by looking at the graphs and mathematically by the 95% limits of agreement. This parameter describes for the user of these perimetry methods, whether clinician or researcher, the magnitude of difference he can expect between each pair of tests in most (95%) instances, excluding the most atypical outliers.

## 4. Discussion

This study presents characterization and comparison of the 10-2 SITA-standard and SITA-fast visual field testing programs. Average test duration was 30% shorter using SITA-fast. A somewhat greater reduction in examination duration of around 40% was reported when the two programs were compared using the 30-2 algorithm [3, 4, 6]. A shorter test duration has the clear advantages of decreasing patient inconvenience and increasing testing efficiency. It is also presumed to potentially improve test reliability through reduction of patient and eye fatigue, but this is not substantiated. There are very few studies that specifically assessed the effect of fatigue on test results in patients with glaucoma, and, in these older, longer-duration algorithms were used [11–13]. In one recent study that evaluated the SITA-standard 24-2 program in patients with glaucoma, changing the order of eye testing (right or left first), did not have a significant effect on test results, suggesting that, on average, single-patient intereye fatigue may not be clinically significant with this algorithm [14].

The 10-2 SITA-standard program yielded slightly more negative values of MD than the 10-2 SITA-fast. A statistically significant difference of 0.3 dB was found in the first set of tests and a smaller nonstatistically significant difference of 0.2 dB in the second set. Similar results among glaucoma patients whereby SITA-standard provides slightly more negative values than SITA-fast have been reported for the 30-2 program [3–5, 7]. In our study, the average number of locations with depressed sensitivity was also similar between the 2 algorithms. In one study evaluating the 30-2 program, it was similarly reported that the number of depressed points was not statistically significantly different between SITA-standard and fast [7]. However, the Bland-Altman analyses showed that significant differences can exist between a given pair of tests using the 2 programs. These observations suggest that, in glaucoma patients experienced with automated perimetry, average results of 10-2 SITA-standard and fast algorithms are similar and may thus be comparable between study populations. However, for the individual patient, if visual field change is suggested by two sequential tests obtained by the different SITA programs, this should be verified by repeat testing.

A similar conclusion is supported by the results of repeated tests with the same program. The test pairs with either SITA-standard or fast yielded average sensitivity results not statistically significantly different, but the Bland-Altman analysis showed that the difference in any given pair may be clinically significant. Thus, even if a single program is used for followup of an individual patient, determination of change likely requires verification, as does establishment of baseline field status. The Bland-Altman analysis showed that SITA-standard had a lower intervisit variability (95% limits of agreement) in mean deviation compared with SITA-fast. However, the two programs had a similar distribution of differences in the number of locations with depressed sensitivity.

The size of our study population was relatively small. However, we think this would present a difficulty in interpretation of results if intertest differences were numerically substantial (and therefore potentially clinically significant). Since in our study the differences in both MD and number of depressed points were minute, then the sample size poses a lesser problem, and achieving statistical significance with such differences would require thousands of members in each study group.

In summary, in a small cohort of patients with advanced glaucoma, we found no or minor differences in the mean results of SITA-standard and SITA-fast 10-2 programs and in repeated testing with each of these programs, implying that test results may be used interchangeably when comparing groups of tests. However, our study also shows that clinically significant differences may exist in a given test pair, both between programs and between visits, and so, in the management of the individual patient, apparent field change should be corroborated by repeat testing.

## Figures and Tables

**Table 1:** Sensitivity parameters (mean ± SD) for each of the 10-2 tests, using 2 programs (SITA-standard and SITA-fast) in 2 separate visits.

	SITA-standard 1	SITA-fast 1	SITA- standard 2	SITA-fast 2
Mean deviation (dB)^1^	−14.8 ± 6.7	−14.5 ± 7.2	−14.3 ± 6.8	−14.1 ± 6.7
Number of depressed points on the pattern deviation plot at least *P* < 2%^2^	34.6 ± 13.5	34.9 ± 12.8	33.4 ± 14.4	33.3 ± 13.4

^1^For each SITA program, results in the 2 visits were not statistically significantly different. Intravisit results for the 2 programs were statistically significantly different during the first (*P* = 0.017) but not the second visit.

^2^For each SITA program, results in the 2 visits were not statistically significantly different. Intravisit results for the 2 programs were not statistically significantly different during both visits.

**Table 2:** Reliability parameters (mean ± SD) for each of the 10-2 tests, using 2 programs (SITA-standard and SITA-fast) in 2 separate visits. There was no statistically significant difference in any parameter for all test pairs.

	SITA-standard 1	SITA-fast 1	SITA- standard 2	SITA-fast 2
Fixation loss (%)	4.7 ± 6.6	8.9 ± 18.3	9.5 ± 13.2	7.3 ± 12.9
False positive (%)	2.1 ± 3.5	2.1 ± 3.2	2.3 ± 4.4	1.4 ± 3.1
False negative (%)	5.3 ± 9.1	3.2 ± 5.4	4.4 ± 6.9	5.9 ± 10.8
